# Double Positive CD4^+^CD8^+^ (DP) T-Cells Display Distinct Exhaustion Phenotype in Chronic Hepatitis C

**DOI:** 10.3390/cells12101446

**Published:** 2023-05-22

**Authors:** Anna Maria Kochanowicz, Sylwia Osuch, Hanna Berak, Aleksandra Kumorek, Kamila Caraballo Cortés

**Affiliations:** 1 Department of Immunopathology of Infectious and Parasitic Diseases, Medical University of Warsaw, 02-106 Warsaw, Poland; 2 Outpatient Clinic, Warsaw Hospital for Infectious Diseases, 01-201 Warsaw, Poland

**Keywords:** CD4^+^CD8^+^ double positive (DP) T-cells, hepatitis C virus, antiviral treatment, DAA, T-cell exhaustion, PD-1, Tim-3

## Abstract

In chronic hepatitis C (CHC), characterized by exhaustion of T-cell function, increased frequencies of double-positive (DP) (CD4^+^CD8^+^) cells are present in peripheral blood. We compared the exhaustion phenotype between DP and single positive (SP) T-cells, including HCV-specific cells, and assessed the effect of successful HCV treatment on inhibitory receptors expression. Blood samples from 97 CHC patients were collected before and six months post-treatment. PD-1 (programmed cell death protein 1) and Tim-3 (T-cell immunoglobulin and mucin domain-containing molecule-3) expression was assessed by flow cytometry. DP T-cells displayed significantly higher PD-1 expression, lower Tim-3 expression than CD8^+^ SP T-cells and lower percentages of PD-1^−^Tim-3^−^ cells than CD4^+^ SP T-cells, both before and after treatment. PD-1^+^Tim-3^+^ DP T-cells decreased following treatment. HCV-specific cells were more frequent among DP than SP T-cells, both before and after treatment. HCV-specific DP T-cells were characterized by lower PD-1 expression, higher PD-1 and Tim-3 co-expression, and lower percentages of PD-1^−^Tim-3^−^ cells (both before and after treatment) and higher post-treatment Tim-3 than HCV-specific SP T-cells. Their percentages decreased following treatment, but the exhaustion phenotype remained unchanged. DP T-cells in CHC exhibit a distinct exhaustion phenotype from SP T-cells, and these changes mostly persist following successful treatment.

## 1. Introduction

Chronic hepatitis C virus (HCV) infection is characterized by functional exhaustion of T-cells, i.e., gradual loss of effector functions and increased susceptibility to apoptosis. This is phenotypically manifested by the increased expression of inhibitory receptors (iRs) on their surface, e.g., PD-1 (programmed cell death protein 1) [1] and Tim-3 (T-cell immunoglobulin and mucin domain-containing protein 3) [2] on total and HCV-specific T-cells [3,4,5,6,7,8]. Increased expression of iRs during an early stage of infection facilitates the development of chronic HCV infection [6,8,9]. Persistent antigen exposure has been recognized as a major driving force of exhaustion, which stimulates iRs expression [3].

Being mostly expressed on activated CD4^+^ and CD8^+^ T-cells, PD-1 represents a marker of early exhaustion, related to impaired proliferation, but still relatively well-preserved T-cell function, including cytokine production [10]. Interaction of PD-1 with its ligands results in the suppression of T-cell sensitivity to antigenic stimulation [11,12].

Tim-3 is regarded to be a marker of more advanced T-cell exhaustion associated with infection progression [2,13], downregulation of IFN-γ production and susceptibility to apoptosis [14,15]. Increased frequencies of Tim-3-expressing CD4^+^ and CD8^+^ T-cells have been observed in chronic HCV infection and were particularly high on HCV-specific CD8^+^ T-cells [2].

Minor frequencies (typically 1–2%) of mature, double positive (DP) T-cells of CD4^+^CD8^+^ phenotype circulate in healthy donors’ peripheral blood [16,17]. Within DP T-cells, typically, two main subpopulations are distinguished: CD4^hi^8^lo^ and CD4^lo^8^hi^, which is determined by the intensity of CD4 and CD8 receptors’ expression. Initially, it was suggested that the DP T-cells are CD4^+^CD8^+^ thymocytes prematurely released into the peripheral blood from the thymus, while others have shown that these are highly specialized cells with antiviral characteristics [18]. Their frequency was shown to be increased during chronic infections such as HCV [19,20], hepatitis B virus (HBV) [19], human immunodeficiency virus (HIV) [21,22,23] or Candida albicans [24], as well as in autoimmune diseases [25] and cancer [26,27]. In these patients, the number of DP T-cells may reach 20% of all circulating lymphocytes [28].

Although DP T-cell function is not fully characterized, they may play a role in adaptive immune responses to infections or tumors since they tend to infiltrate a site of chronic infection or tumor lesion [19,28]. For example, higher frequencies of DP T-cells are found in liver than in peripheral blood of patients with chronic hepatitis C (CHC) [19,28]. Although little is known about the origin of DP T-cells, a recent study indicates that in patients with HCC, these may be derived from intra-tumor single positive (SP) CD8^+^ T-cells [27].

We have previously demonstrated similar frequencies of DP T-cells in patients with CHC and in patients with past infection, and these were both higher than in HCV-negative controls [20]. Furthermore, percentages of PD-1-expressing DP T-cells were higher than single positive (SP) PD-1^+^ T-cells [20]. However, that study was performed on a relatively small group of patients.

Recently, a highly effective antiviral treatment based on direct acting antivirals (DAA) has been developed to be used in chronic HCV infection, making viral eradication achievable in nearly all treated patients [29,30]. Still, very little is known about the effect of DAA-based treatment-driven viral eradication on the parameters of T-cell exhaustion of DP T-cells, including reversibility of this immune-related phenomenon.

The aim of the study was to determine the exhaustion phenotype of DP T-cells when compared to SP T-cells in CHC and to investigate whether DP T-cells’ exhaustion phenotype was altered following HCV elimination by means of DAA-based treatment.

## 2. Materials and Methods

Ninety-seven patients with a diagnosis of chronic hepatitis C infection (anti-HCV^+^, HCV RNA^+^) were recruited from the Warsaw Hospital for Infectious Diseases right before and six months following completion of HCV treatment with DAAs according to the standard recommendations. All patients were HCV RNA positive for at least six months prior to therapy. Inclusion criteria were infection with genotype 1b, no evidence of cirrhosis and no other potential cause of chronic liver disease.

HCV genotype was assessed by Inno-LiPA HCV II (Innogenetics). Baseline liver fibrosis scores were assessed by transient elastography [31]. A quantitative PCR test (Abbott RealTime HCV Viral Load Assay, Abbott Laboratories; sensitivity 12 IU/mL) was used to evaluate baseline viral load as well as clinical effectiveness of treatment (assessed 6 months post-treatment). Sustained virologic response (SVR) was achieved in all patients (100%). Some clinical and epidemiological characteristics of the study participants are presented in Table 1.

The study protocol followed ethical guidelines of the 2013 Declaration of Helsinki and was approved by the Bioethical Committee of the Medical University of Warsaw (KB/77/A/2015) and all patients provided written informed consent.

A total of 10 mL of EDTA-anticoagulated blood was obtained by venipuncture, from which peripheral blood mononuclear cells (PBMCs) were isolated by density gradient centrifugation using Lymphoprep (Stemcell Technologies Inc, Vancouver, BC, Canada). Cells were counted and four million were resuspended in PBS solution (Life Technologies, Carlsbad, CA, USA) and BD Horizon Fixable Viability Stain 780 (BD Biosciences, San Diego, CA, USA), incubated for 15 min at room temperature and protected from light exposure. After washing twice in 2 mL of BD Pharmingen Stain Buffer (BD Biosciences), cells were incubated for 15 min at room temperature with FcR blocking reagent (Miltenyi Biotec, Bergisch Gladbach, Germany).

**Figure 1:**
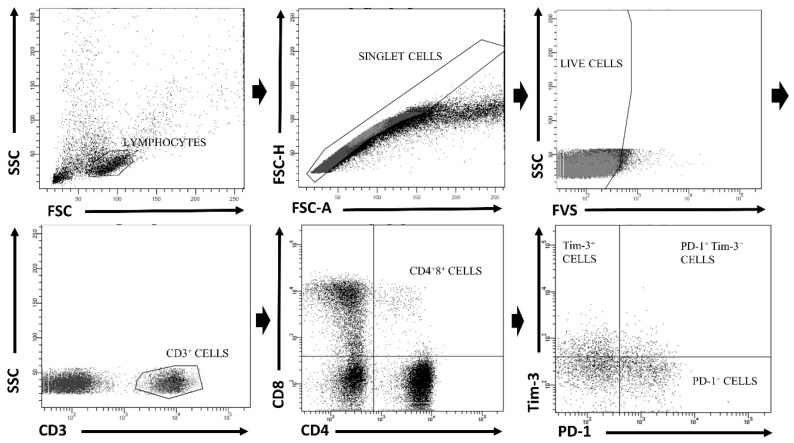
Gating strategy of flow cytometric phenotyping of double positive (DP) (CD3^+^CD4^+^CD8^+^) T-cells.

One million of cells were then stained with BD Horizon BV421 Mouse Anti-Human Tim-3 (CD366), BD Pharmingen Alexa Fluor 647 Mouse Anti-Human PD-1 (CD279), BD Pharmingen PerCP-Cy 5,5 Mouse Anti-Human CD3, BD Horizon V500 Mouse Anti-Human CD4 and Proimmune FITC Mouse Anti-Human CD8 for 20 min at 4 °C. Controls comprised 1 million of unstained cells and fluorescence minus one (FMO) controls, in which BD Pharmingen Alexa Fluor 647 IgG1 κ and BV421 IgG1 κ were used instead of anti-PD-1 and anti-Tim-3, respectively. After washing twice with PBS, cells were immediately analyzed using a BD FACS Canto II flow cytometer (Becton Dickinson) and BD FACS Diva version 6.0 software (Becton Dickinson). For the purpose of data analysis, an initial lymphocyte gate was set based on forward scatter (FSC)/side scatter (SSC); then, cell aggregates were excluded based on the FSC-A vs. FSC-H parameters. Subsequently, dead cells (FVS 780^−^) were gated out and the percentages of CD3^+^CD4^+^, CD3^+^CD8^+^ and CD3^+^CD4^+^CD8^+^ T-cells with PD-1 and/or Tim-3 expression were assessed. The employed gating strategy is presented in Figure 1.

For HCV-specific SP and DP T-cells analysis, we employed additional staining of cells specific to HLA-A*A02-restricted KLVALGLNAV peptide in HLA-A*A02-positive subjects using a custom PE-conjugated MHC pentamer (ProImmune, Oxford, United Kingdom) combined with enrichment of these cells using anti-PE magnetic beads as previously described [32,33]. Enriched cells were subjected to analogic surface antibody staining. Exhaustion phenotype was assessed only if the number of HCV-specific SP/DP T-cells was higher or equal than *n* = 10.

Results were presented as mean values ± standard error or median (range). To compare the frequencies of DP T-cells, as well as the percentages of these cells with exhaustion markers expression before and after treatment, Wilcoxon matched-pairs signed-ranks test was used. The differences between percentages of CD4^hi^CD8^lo^ and CD4^lo^CD8^hi^ T-cells were assessed using the Mann–Whitney Test. Kruskal–Wallis test was used to compare percentages of CD3^+^CD4^+^, CD3^+^CD8^+^ SP and CD3^+^CD4^+^CD8^+^ DP T-cells with exhaustion markers’ expression. All *p*-values were two-tailed and considered significant when lower than 0.05.

## 3. Results

### 3.1. The Impact of Successful Treatment on the Percentages of Total DP T-Cells

**Figure 2:**
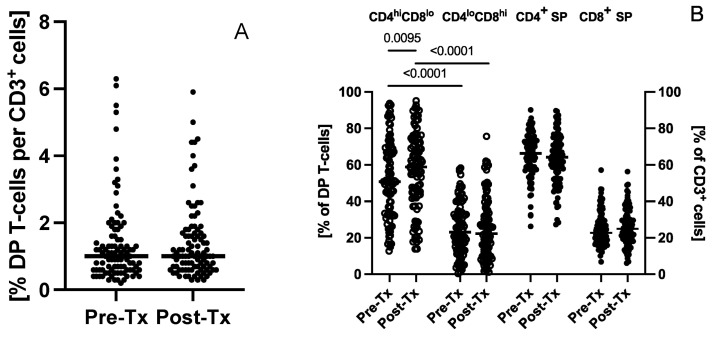
Successful HCV treatment effect on percentages of T-cells of double positive (CD3^+^CD4^+^CD8^+^) (**A**) as well as CD4^hi^CD8^lo^, CD4^lo^CD8^hi^, SP CD3^+^CD4^+^ and SP CD3^+^CD8^+^ (**B**) phenotype. Pre-Tx—before treatment; Post-Tx—after treatment. Horizontal lines within each population of results represent median values. Numbers above lines representing statistical comparisons express *p* values.

DP T-cells constituted 1.4 ± 0.1, 1.0 (0.2–6.3)% (mean ± SE, median (range)) of CD3^+^ cells. There was no significant change in the frequencies of DP T-cells in patients following successful treatment (1.4 ± 0.1, 1.0 (0.3–5.9)% of CD3^+^ cells) (Figure 2A). Among DP T-cells, CD4^hi^CD8^lo^ cells were significantly more frequent than CD4^lo^CD8^hi^ cells, both before (50.8 (12.7–93.7) vs. 23.0 (0.4–58.3)%, *p* < 0.0001) and after (58.8 (13.8–95.1) vs. 22.3 (1.1–75.6)%, *p* < 0.0001) treatment (Figure 2B). The ratio of CD4^hi^CD8^lo^/CD4^lo^CD8^hi^ was 2.2 and 2.6 before and after treatment, respectively. Furthermore, the percentage of CD4^hi^CD8^lo^ T-cells has significantly increased after treatment (*p* = 0.0095), while the percentage of CD4^lo^CD8^hi^ T-cells did not significantly change (Figure 2B).

CD4^+^ SP and CD8^+^ SP T-cell frequency did not change after treatment (from 66.3 (26.2–90.1) to 64.2 (27.2–89.7)% for CD4^+^ cells and from 22.7 (6.8–57.1) to 24.9 (6.0–58.3)% for CD8^+^ cells). The median ratio of CD4/CD8 cells was 2.9 and 2.6 before and after treatment, respectively.

### 3.2. Peripheral DP T-Cells Are Characterized by a Distinct Exhaustion Phenotype from Their SP Counterparts

**Figure 3:**
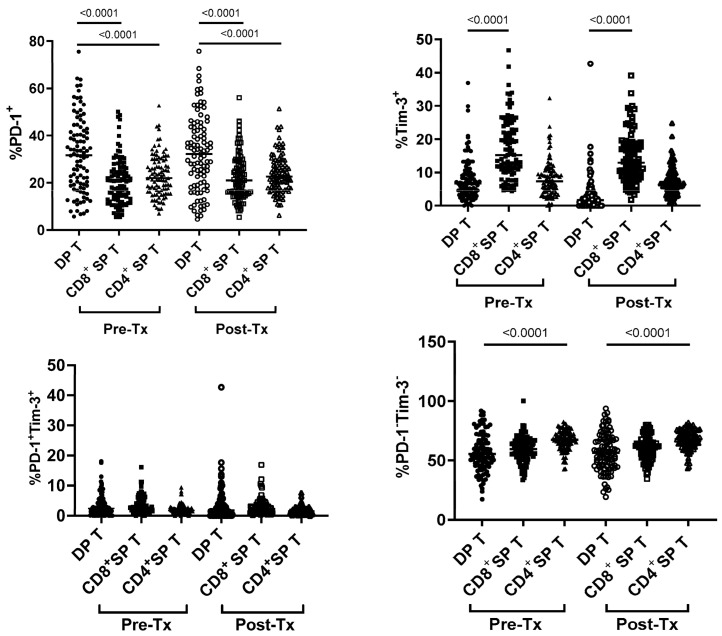
Percentages of T-cells of DP CD3^+^CD4^+^CD8^+^, SP CD3^+^CD4^+^ and SP CD3^+^CD8^+^ phenotype expressing exhaustion markers. Pre-Tx—before treatment; Post-Tx—after treatment. Horizontal lines within each population of results represent median values. Numbers above lines representing statistical comparisons indicate *p* values.

There were significant differences between SP and DP T-cells in exhaustion markers’ expression (Figure 3). The highest percentage of PD-1^+^ cells was observed among DP when compared to CD4^+^ and CD8^+^ SP T-cells, both before (31.6 (5.7–75.5) vs. 22.0 (7–52.7) vs. 20.7 (5.5–50.1)%, respectively, *p* < 0.0001)) and after treatment (32.2 (4.5–75.7) vs. 22.6 (6.2–51.3) vs. 21 (5.4–56.0)%, respectively (*p* < 0.0001)). In contrast, the percentages of cells expressing Tim-3 were significantly lower among DP T-cells than among SP CD8^+^ T-cells, but not among CD4^+^ SP T-cells, both before (6.7 (0.0–36.9) vs. 15.2 (4.3–46.7) *p* < 0.0001 vs. 7.3 (0.0–32.3)%, respectively) and after treatment (5.8 (0–44.4) vs. 12.9 (1.8–39.1), *p* < 0.0001 vs. 6.4 (0.7–24.8)%, respectively). There were no significant differences between DP and CD4^+^ and CD8^+^ SP T-cell percentages co-expressing PD-1 and Tim-3, both before (2.4 (0.0–18.1) vs. 1.7 (0.0–9.4) vs. 2.5 (0.0–16.1)%, respectively) and after treatment (1.7 (0.0–42.7) vs. 1.5 (0.0–7.6) vs. 1.9 (0.2–16.9)%, respectively). In contrast, DP T-cells expressing neither PD-1 nor Tim-3 were significantly lower than CD4^+^ SP T-cells, but not CD8^+^ T-cells, both before (55.3 (17.2–91.7) vs. 67.3 (42.6–82.4) (*p* < 0.0001) vs. 59.3 (33.6–79.4)%, respectively) and after treatment (56.4 (19.0–93.7) vs. 67.6 (43.4–82.0) *p* < 0.0001) vs. 60.9 (34.3–80.1)%, respectively).

### 3.3. Successful Treatment Reduces Percentages of DP T-Cells Co-Expressing PD-1 and Tim-3

**Figure 4:**
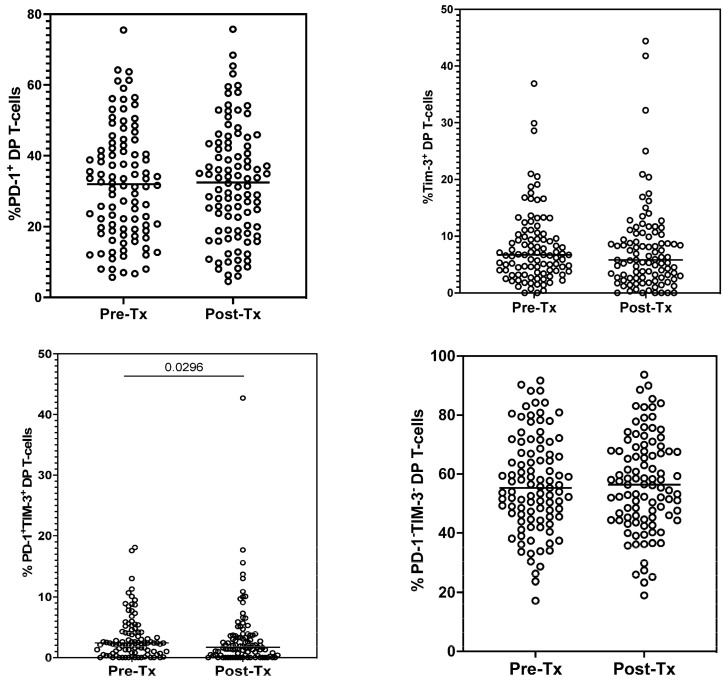
Percentages of T-cells of DP CD3^+^CD4^+^CD8^+^ phenotype expressing exhaustion markers before (Pre-Tx) and after (Post-Tx) HCV treatment. Horizontal lines within each population of results represent median values. Numbers above lines representing statistical comparisons indicate *p* values.

The percentage of DP T-cells expressing either PD-1 or Tim-3 did not change significantly following treatment (from 31.6 (5.7–75.5) to 32.2 (4.5–75.7)% and from 6.7 (0.0–36.9) to 5.8 (0–44.4)% for PD-1 and Tim-3, respectively) (Figure 4). Similarly, there were no statistically significant differences in the percentages of DP T-cells with neither PD-1 nor Tim-3 expression (55.3 (17.2–91.7)% vs. 56.4 (19.0–93.7)%). However, the percentage of DP T-cells co-expressing PD-1 and Tim-3 significantly decreased after treatment (from 2.4 (0.0–18.1) to 1.7 (0.0–42.7)%, *p* = 0.0296) (Figure 4).

### 3.4. HCV-Specific DP T-Cells Were Characterized by Distinct Exhaustion Phenotype from Their SP Counterparts

**Figure 5:**
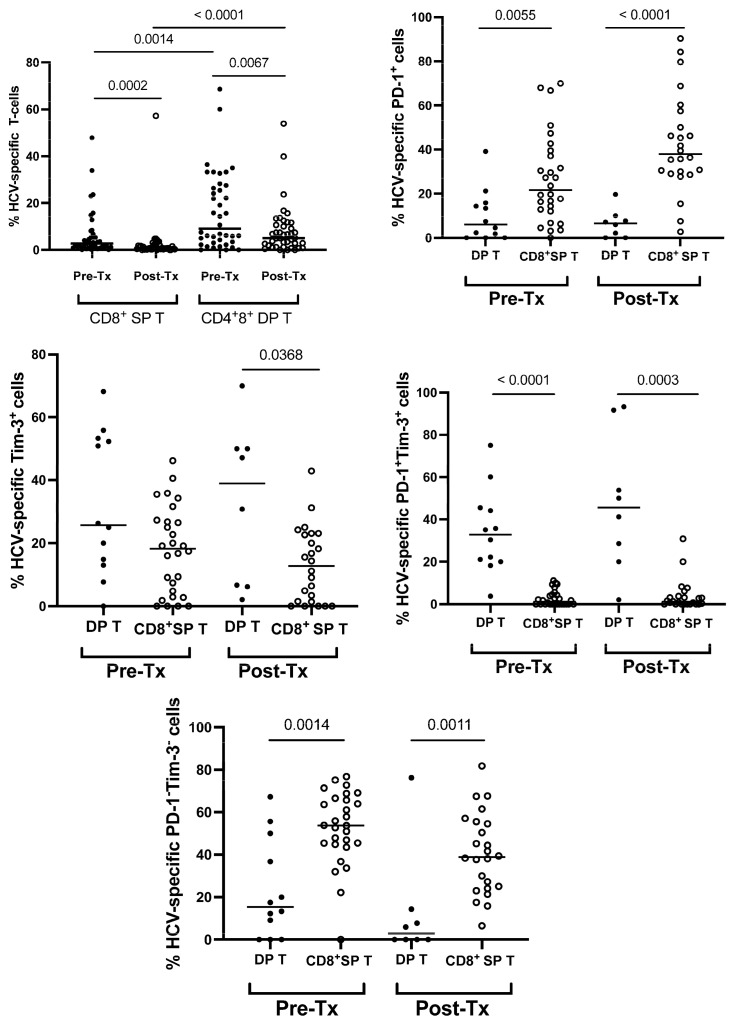
Percentages of HCV-specific T-cells of DP CD3^+^CD4^+^CD8^+^ and SP CD3^+^CD8^+^ phenotype expressing exhaustion markers. Pre-Tx—before treatment; Post-Tx—after treatment. Horizontal lines within each population of results represent median values. Numbers above lines representing pairwise comparisons indicate *p* values.

**Figure 6:**
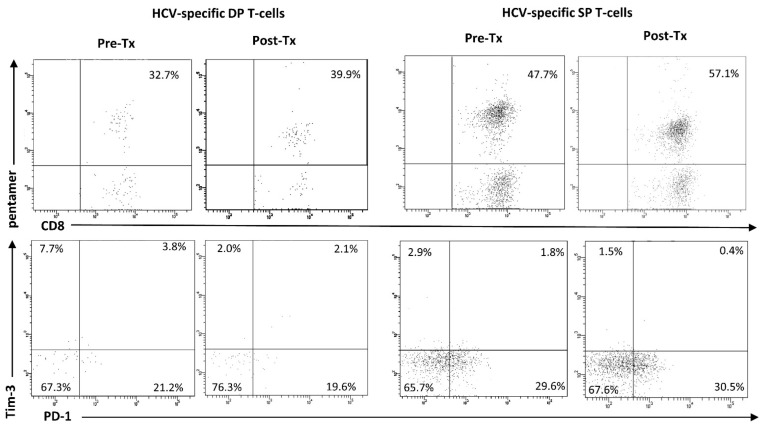
Representative cytometric analysis of percentages of HCV-specific DP T-cells and SP T-cells as well as their PD-1/Tim-3 expression phenotype before and after successful treatment in Patient 30 (Pt_30). Pre-Tx before treatment, Post-Tx after treatment.

At baseline, HCV-specific T-cells were significantly more frequent among DP than SP cells (9.0 (0.0–68.6) vs. 2.7 (0.1–47.9)%, *p* = 0.0014). HCV-specific DP T-cells remained more frequent than HCV-specific SP T-cells after treatment (5.1 (0.0–53.9) vs. 0.7 (0.0–57.2)%, *p* < 0.0001) (Figure 5). Furthermore, HCV-specific DP T-cells were characterized by significantly lower PD-1 expression, both before (5.9 (0.0–39.1)%, vs. 21.6 (0.0–70.0)%, *p* = 0.0055) and after treatment (6.5 (0.0–19.6)% vs. 37.9 (2.7–90.3)%, *p* < 0.0001); higher post-treatment Tim-3 (38.9 (2.1–70.0)% vs. 12.7 (0.0–42.9)%, *p* = 0.0368); higher PD-1 and Tim-3 co-expression, both before (32.7 (3.8–75.0)% vs. 1.2 (0.0–11.1)%, *p* < 0.0001) and after treatment (45.6 (2.1–93.3)% vs. 1.1 (0.0–30.8)%, *p* = 0.0003); and lower percentages of cells with neither PD-1 nor Tim-3 expression, both before (15.3 (0.0–67.3)% vs. 53.7 (0.0–76.8)%, *p* = 0.0014) and after treatment (2.9 (0.0–76.3)% vs. 38.9 (6.5–81.8)%, *p* = 0.0011) than SP counterparts (Figure 5). Representative cytometric analyses of percentages of HCV-specific DP T-cells and SP T-cells, as well as their PD-1/Tim-3 expression phenotype before and after successful treatment, are presented in Figure 6.

### 3.5. The Impact of Successful Treatment on the Percentages of HCV-Specific DP T-Cells

Similar to HCV-specific SP cells, HCV-specific DP T-cells’ percentages decreased following successful treatment (from 2.7 (0.1–47.9) vs. 0.7 (0.0–57.2), *p* = 0.0002 and from (9.0 (5.1–68.6) to 5.1 (0.0–53.9), *p* = 0.0067, respectively). However, there were no significant differences in the percentages of HCV-specific DP T-cells with PD-1, Tim-3 or PD-1+Tim-3 expression, or with neither PD-1 nor Tim-3 expression (Figure 5).

## 4. Discussion

The presented study aimed to determine the exhaustion phenotype of DP T-cells when compared to SP T-cells in CHC and to investigate whether expression of PD-1 and/or Tim-3 on DP T-cells was altered following HCV elimination by means of DAA-based treatment. Viral eradication may hypothetically be related to the reversal of the exhaustion phenotype due to elimination of HCV antigen burden, including viral proteins known to inhibit immune responses and reduction in the production of immunosuppressive cytokines, e.g., IL-10, which has been recognized as being directly stimulated by HCV [34,35,36,37]. With the advent of highly successful DAA-based treatment schemes, it became uniquely possible to study the effect of viral eradication on T-cell exhaustion phenotype on already established chronic HCV infection; however, so far, this aspect has not been studied in the context of DP T-cells. This is especially valid given that these cells are suspected to be directly involved in immune responses in chronic HCV infection, and that eventual treatment-driven renewal of these cells may be important for boosting anti-HCV immunity.

To accomplish these goals, we recruited a large cohort of chronically HCV-infected patients, uniform in terms of the infecting genotype (G1), and employed a comprehensive cytometric assessment of two inhibitory receptors, i.e., PD-1 and Tim-3, including their dual expression, which was previously shown to be associated with more profound exhaustion than single expression [3,38]. Importantly, our analysis comprised phenotypic assessment of not only total DP T-cells but also HCV-specific DP T-cells, known to be difficult to be detected directly in vivo in peripheral blood during chronic infection [39,40].

Our study revealed that the mean frequency of peripheral DP T-cells was 1.4% of CD3^+^ T-cells, close to those reported by Nascimbeni et al. [28], who found the frequency of DP T-cells in HCV-infected patients to be 1.2%. Importantly, no change in their frequency was observed in our study upon HCV antigen elimination (post-treatment). This was also shown in our previous cross-sectional analysis in which a similar frequency of DP T-cells in CHC patients and in subjects who spontaneously cleared the virus was observed [20]. Noteworthy, the frequency of DP T-cells largely varied between the patients, which suggests that the increased frequency of DP T-cells may be of a transient nature.

Our study revealed two distinct subpopulations of DP T-cells: CD4^hi^8^lo^ and CD4^lo^8^hi^, of which the former significantly prevailed over the latter and the relative proportions of CD4^hi^CD8^lo^/CD4^lo^CD8^hi^ were 2.2 and 2.6 before and after treatment, respectively. This is in concordance with the previous findings in which the CD4^hi^8^lo^/CD4^lo^8^hi^ ratio depended on the infection outcome and was higher when infection was controlled and decreased upon progression to chronic infection [24]. Furthermore, these ratios tended to be similar to CD4^+^/CD8^+^ SPT-cells in peripheral blood, which may imply that CD4^hi^8^lo^ and CD4^lo^8^hi^ may originate from their respective SP cell subpopulations in CHC. Similar prevalence of CD4^hi^8^lo^ over CD4^lo^8^hi^ DP T-cells during chronic HCV infection was observed in our previous study [20] as well as in other studies [19,23]. Thus, CD4^hi^8^lo^ DP T-cells are likely to be generated during chronic immune processes. Interestingly, CD4^hi^8^lo^ percentage increased post-treatment, while CD4^lo^8^hi^ cells remained stable, which suggests a rather distinct survival characteristic of these cells. This may be related to the finding of Nascimbeni et al., in which more than a half of the peripheral CD4^lo^CD8^hi^ DP T-cells expressed CCR7, a marker of naïve and memory T-cells, which was detected much less often on CD4^hi^CD8^lo^ DP T-cells in the periphery during chronic HCV infection [19].

Noteworthy, the study revealed that total peripheral blood DP T-cells were characterized by a distinct exhaustion phenotype from their SP counterparts, which was congruent with our previous study [20]. Interestingly, the history of a previous unsuccessful treatment did not have an impact on baseline exhaustion phenotype of the analyzed cells. All the above differences in exhaustion phenotype between DP and SP T-cells were maintained following treatment-driven HCV eradication, which implies that once generated during chronic infection, the relative status quo is preserved for at least for six months post-treatment. This also suggests that the treatment-driven changes affecting both populations seem to be symmetrical.

Importantly, we demonstrated that successful HCV-oriented treatment is associated with a change of DP T-cells exhaustion phenotype, manifesting in a reduction in proportions of PD-1^+^Tim-3^+^ DP T-cells. The observed differences were not likely caused by changes in percentages of these cells themselves, as these remained stable. Similarly, the differences in treatment scheme and the fact of previous treatment did not have an impact on the observed effects.

It was previously found that co-expression of PD-1 and Tim-3 characterizes terminally differentiated, exhausted T-cells [3,38,41,42,43,44,45]. Interestingly, co-expression patterns are functionally related, as a concurrent blocking of these multiple co-inhibitory receptors leads to synergistic reversal of exhaustion [3,38,46]. This suggest a renewal of the DP T-cell population following viral eradication in terms of exhaustion, which could be due to DP becoming either PD-1^+^ or Tim-3^+^ or deleted. A similar observation was made previously for SP cells, in case of which DAA-treatment resulted in a significant decrease in CD4^+^PD-1^+^Tim-3^+^ and CD8^+^PD-1^+^Tim-3^+^ SP T-cell frequencies to levels observed in controls [32].

Several studies addressed the question how DAA-achieved viral eradication impacts the exhaustion phenotype of SP T-cells, but not DP T-cells. Although the collected data are largely fragmentary and sometimes even contradictory, most of them provided evidence that DAA treatment may result in at least partial restoration of SP T-cell immune function as evidenced by an increase in frequency of CD4^+^ [47,48,49] and CD8^+^ T-cells [48] and a shift toward T_em_ (effector memory) population [47,50], with a concomitant decrease in the naïve T-cell subset [50]. Effector function reinvigoration was also observed, manifested by increased frequencies of circulating T helper and cytotoxic T-cells producing IFN-γ, IL-17 and IL-22 [51]. Furthermore, a reduction of PD-1 [50] and TIGIT [47,49,50] expression on both CD4^+^ and CD8^+^ SP T-cells were reported.

Our in-depth insight into the HCV-specific DP T-cells subset has allowed us to characterize the frequency as well as, for the first time, the exhaustion phenotype of these cells. Importantly, HCV-specific DP T-cells were found to be significantly more frequent among HCV-specific DP than SP T-cells, both before and after treatment. This implies that HCV-specific DP T-cells play a pivotal, perhaps even critical role in anti-HCV immunity. Indeed, one study has shown that the frequency of HCV-specific cytokine-producing cells upon HCV antigen stimulation was markedly higher among the DP than the SP populations [28].

Our study demonstrated that HCV elimination following successful treatment was associated with a decrease in frequency but not complete deletion of both HCV-specific DP and SP T-cells cell subsets, which suggests a memory phenotype as well as the antigen-independent character of these cells. The common central effector memory phenotype of DP T-cells was formerly demonstrated by the expression of CCR7, CD45RO, CXCR3, CCR6 and activation markers CD57 and CD95 [21,23,25,28,52]. These cells also displayed higher antiviral activity than SP T-cells, manifesting in a higher proliferative capacity and increased interferon γ (IFN-γ), tumor necrosis factor α (TNF-α), IL-2, IL-4, IL-10, IL-17A and lytic enzymes’ production upon antigen stimulation [21,25,52]. Furthermore, the percentage of cells with differentiated phenotypes, represented by lower T-cell receptor excision circles (TREC) content and shorter telomeres, was shown to be higher among DP than single-positive (SP) T-cells [28].

Importantly, HCV-specific DP T-cells were characterized by a distinct pattern of PD-1/Tim-3 expression, implying higher exhaustion of these cells, possibly due to antigen overstimulation and triggering, again pointing to their involvement in HCV-specific immunity. For comparison, in HIV-1 infection, the frequency of DP T-cells as well as the expression of activation and exhaustion markers on these cells increased as the infection progressed [23,53]. Interestingly, the observed differences were mostly retained between pre- and post-treatment, which implies that the exhaustion phenotype of HCV-specific DP T-cells is poorly modifiable once the “offending” pathogen is eradicated. A similar observation we made previously in the case of SP HCV-specific T-cells [32].

Despite being the first of its kind, our study also has shortcomings. First of all, our observations are limited to the peripheral T-cells, which may display a distinct exhaustion phenotype from intrahepatic T-cells. Since liver biopsy is no longer a standard for a liver disease stage evaluation prior to CHC treatment, we were unable to address this issue.

Second, the study aimed at a characterization of DP T-cells’ phenotype rather than their functional features. Nevertheless, because of the employment of multiparametric flow cytometry, we were able to capture not only a single expression but also co-expression of iRs, which was previously shown to largely determine exhaustion. Delineating the exact role of DP T-cells in HCV immunity will require further research, in particular, analysis of not only the exhaustion phenotype but also their transcriptomic and functional features.

## 5. Conclusions

In summary, the results of the study provided an insight into the exhaustion phenotype of DP T-cells in chronic HCV infection and the effect of successful treatment. We found that total peripheral DP T-cells were characterized by a distinct exhaustion phenotype from their SP counterparts, both before and after treatment. Successful HCV treatment had an impact on DP T-cells exhaustion, pronounced in a reduction in proportions of PD-1^+^Tim-3^+^ DP T-cells and suggesting of a renewal of this population. HCV-specific cells were more frequent among DP than SP T-cells and were characterized by higher exhaustion, both before and after treatment. Their percentages decreased following successful treatment, but the exhaustion phenotype remained unchanged. Our findings suggest that DP T-cells play an important, perhaps even critical role in the immune response to HCV.

## Figures and Tables

**Table 1:** Clinical and epidemiological characteristics of the study participants.

Sex (female/male)	62/35
Median age (range) [years]	58 (28–83)
Genotype 1b/Genotype 1a	95/2
Baseline median viral load (range) [IU/mL]	8.3 × 10^5^ (6.2 × 10^3^–1.1 × 10^7^)
Treatment scheme	Ledipasvir + Sofosbuvir, *n* = 69 Ombitasvir, Paritaprevir, Ritonavir + Dazabuvir, *n* = 21 Elbasvir + Grazoprevir, *n* = 7
Baseline liver fibrosis stage	F0/1 *n* = 56 F2 *n* = 27 F3 *n* = 14
Previous unsuccessful treatment history	*n* = 24

## Data Availability

The data presented in this study are available on request from the corresponding author. The data are not publicly available due to ethical restrictions.
